# Relationship between jejunum ATPase activity and antioxidant function on the growth performance, feed conversion efficiency, and jejunum microbiota in Hu sheep (*Ovis aries*)

**DOI:** 10.1186/s12917-024-04100-0

**Published:** 2024-06-04

**Authors:** Zhanyu Chen, Guoxiu Wang, Weimin Wang, Xiaojuan Wang, Yongliang Huang, Jiale Jia, Qihao Gao, Haoyu Xu, Lijuan He, Yunfei Xu, Zhen Liu, Jinlin Sun, Chong Li

**Affiliations:** 1 College of Animal Science and Technology, Gansu Agricultural University, Lanzhou, Gansu 730070 China; 2 The State Key Laboratory of Grassland Agro-ecosystems, College of Pastoral Agriculture Science and Technology, Lanzhou University, Lanzhou, Gansu 730020 China; 3 Gansu Runmu Bio-Engineering Co.,LTD, Yongchang, Gansu 737200 China

**Keywords:** Jejunum, ATPase, MDA, Antioxidant function, Feed conversion ratio

## Abstract

**Background:**

ATPase activity and the antioxidant function of intestinal tissue can reflect intestinal cell metabolic activity and oxidative damage, which might be related to intestinal function. However, the specific influence of intestinal ATPase activity and antioxidant function on growth performance, feed conversion efficiency, and the intestinal microbiota in sheep remains unclear.

**Results:**

This study analyzed the correlation between ATPase activity and antioxidant function in the jejunum of 92 Hu sheep and their growth performance and feed conversion efficiency. Additionally, individuals with the highest (H group) and lowest (L group) jejunum MDA content and Na^+^ K^+^-ATPase activity were further screened, and the effects of jejunum ATPase activity and MDA content on the morphology and microbial community of sheep intestines were analyzed. There was a significant correlation between jejunum ATPase and SOD activity and the initial weight of Hu sheep (*P* < 0.01). The H-MDA group exhibited significantly higher average daily gain (ADG) from 0 to 80 days old and higher body weight (BW) after 80 days. ATPase and SOD activities, and MDA levels correlated significantly and positively with heart weight. The jejunum crypt depth and circular muscle thickness in the H-ATP group were significantly higher than in the L-ATP group, and the villus length, crypt depth, and longitudinal muscle thickness in the H-MDA group were significantly higher than in the L-MDA group (*P* < 0.01). High ATPase activity and MDA content significantly reduced the jejunum microbial diversity, as indicated by the Chao1 index and observed species, and affected the relative abundance of specific taxa. Among species, the relative abundance of *Olsenella umbonata* was significantly higher in the H-MDA group than in the L-MDA group (*P* < 0.05), while *Methanobrevibacter ruminantium* abundance was significantly lower than in the L-MDA group (*P* < 0.05). *In vitro* culture experiments confirmed that MDA promoted the proliferation of *Olsenella umbonata*. Thus, ATPase and SOD activities in the jejunum tissues of Hu sheep are predominantly influenced by congenital factors, and lambs with higher birth weights exhibit lower Na^+^ K^+^-ATPase, Ca^2+^ Mg^2+^-ATPase, and SOD activities.

**Conclusions:**

The ATPase activity and antioxidant performance of intestinal tissue are closely related to growth performance, heart development, and intestinal tissue morphology. High ATPase activity and MDA content reduced the microbial diversity of intestinal tissue and affect the relative abundance of specific taxa, representing a potential interaction between the host and its intestinal microbiota.

**Supplementary Information:**

The online version contains supplementary material available at 10.1186/s12917-024-04100-0.

## Background

Intensive confinement feeding of sheep offers several advantages, including the potential for efficient space utilization, optimization of the sheep’s nutritional intake through formulated diets, and the capacity of intensive systems to provide more predictable and consistent production levels, which can be crucial for meeting market demands [1, 2]. However, several studies have indicated that intensive farming can induce a stress response, leading to adverse effects on feed intake, growth, and gastrointestinal health. This stress response can increase intestinal pressure and ultimately impact the incidence of diarrhea [3–5], resulting in economic setbacks and higher breeding expenses. Additionally, intensive farming significantly increased feed costs, which is an important factor affecting economic benefit. Therefore, improving feed conversion efficiency and growth performance under indoor feeding conditions is crucial. In recent years, research on improving the feed conversion efficiency of sheep meat has mainly included the selection of fattening sheep with higher feed reward, the regulation of the feed formula and raw material selection, the improvement of gastrointestinal function, and intervention to alter the gastrointestinal microflora [6–8]. These studies have shown that the feed conversion efficiency of sheep meat is influenced by genetics, nutrition, gastrointestinal development, and the microbial flora [9–11].

The small intestine is an important constituent of the feed digestion and absorption process, absorbing most of the body’s protein, sugar, and fat [12, 13]. Among various cell types, intestinal epithelial cells display exceptionally high metabolic activity, primarily because of their integral role in nutrient absorption. They require energy to transport nutrients across the cell membrane, maintain ion gradients, and perform various metabolic processes [14, 15]. The energy metabolism of intestinal epithelial cells is finely tuned to support their critical role in nutrient absorption and transport. These cells prioritize glucose utilization and rely on mitochondria to produce adenosine triphosphate (ATP) through glycolysis and oxidative phosphorylation [16, 17]. However, this metabolic process also generates an increased amount of oxygen free radicals. To date, there have been few reports on the effects of intestinal energy metabolism and antioxidant function on the feed conversion efficiency of Hu sheep.

ATPase is a critical enzyme found in various cellular membranes and organelles. Its primary function is to catalyze the hydrolysis of ATP molecules into adenosine diphosphate (ADP) and inorganic phosphate (Pi). This enzymatic reaction releases energy that can be harnessed for various cellular processes. In addition, many ATPase enzymes, such as the Na^+^ K^+^-ATPase and Ca^2+^ Mg^2+^-ATPase, are involved in active transport processes across cell membranes. This ion pumping is crucial for cell membrane potential and nerve cell function. At the same time, Na^+^ K^+^-ATPase and Ca^2+^ Mg^2+^- ATPase can protect the heart and lung and reduce myocardial damage [18–20]. Studies have shown that a reduction in ATPase will damage the function of the body’s sodium potassium pump and calcium and magnesium pump, and the ATPase in intestinal tissues can directly affect the energy metabolism and functional damage of animal intestinal tissues [21, 22]. In the production of large amounts of energy (ATP), the body will produce a various of free radicals. The gastrointestinal (GI) tract is the key source of reactive oxygen species (ROS) [23]. When too many free radicals accumulate in the body, they will induce a stress response, which will adversely affect the development of the digestive tract, resulting in decreased feed intake. In addition, the stimulation of these stress factors will be transmitted into the brain through the nervous system, causing disorders of the endocrine system. Studies have also shown that acute or chronic stress in animals can induce gastrointestinal oxidative stress through the production of free radicals, resulting in intestinal damage, intestinal dysfunction, or intestinal flora disturbance, which will change intestinal permeability and affect the intestinal mucosal barrier function [24–26]. In addition, stress-induced free radicals produced by the body will attack the unsaturated fatty acids in biofilms, triggering lipid peroxidation, and thus forming lipid peroxides, such as malondialdehyde (MDA). MDA, which is very harmful to the body, is the end product of lipid peroxidation *in vivo*, and can directly or indirectly reflect the degree of lipid peroxidation and cell damage in the body. MDA can cross-link with proteins and enzymes to affect metabolic function [27]. It’s dysregulation is one of the main causes of metabolic disorders [28, 29]. The MDA content in jejunum tissue can directly or indirectly reflect the intensity and rate of lipid peroxidation in the intestine, and the degree of intestinal tissue damage. When the body produces too many free radicals, the MDA content increases and the body’s anti-damage ability decreases; On the contrary, when the body produces few free radicals, the MDA content decreases and the body’s ability to resist damage is improved [30–32]. There are a few reports that MDA may act as a signal messenger to regulate gene expression [33]; however, its biological function and dual role have not been fully studied.

Therefore, the ATPase activity and antioxidant function of intestinal tissue can reflect the metabolic activity and oxidative damage of intestinal cells, which might be closely related to intestinal function and the feed conversion efficiency. Consequently, it is necessary to improve animal growth traits and their feed conversion efficiency by intervening with the energy metabolism process of intestinal cells and ensuring the balance of the intestinal redox state via nutrient regulation. However, the relationship between intestinal ATPase activity and antioxidant function and intestinal function and feed conversion efficiency, and its regulatory mechanism, remain unclear. The jejunum is the longest segment of the small intestine and plays a crucial role in nutrient absorption and digestion. Therefore, we hypothesized that jejunal ATPase activity and antioxidant function of sheep regulate jejunal development and function, thereby affecting the growth performance and feed conversion efficiency. This would influence the jejunal microbiota through host-microbial interactions. To test these hypotheses, herein, the long-term growth traits and feed conversion efficiency of 92 Hu sheep were measured, and the ATPase activity, antioxidant function, and intestinal morphology of jejunum tissues were determined after slaughter at 180 days old. We aimed to analyze the effects of intestinal ATPase activity and antioxidant function on important economic traits and intestinal functions of sheep. 16s rRNA amplicon sequencing was used to investigate the regulation of antioxidant function on the jejunal microbiota, and the host-microbial interaction was further verified by anaerobic culture of specific microorganisms.

## Results

### Correlation analysis of intestinal ATPase activity and antioxidant indexes with growth traits and feed conversion efficiency of Hu sheep

**Fig. 1:**
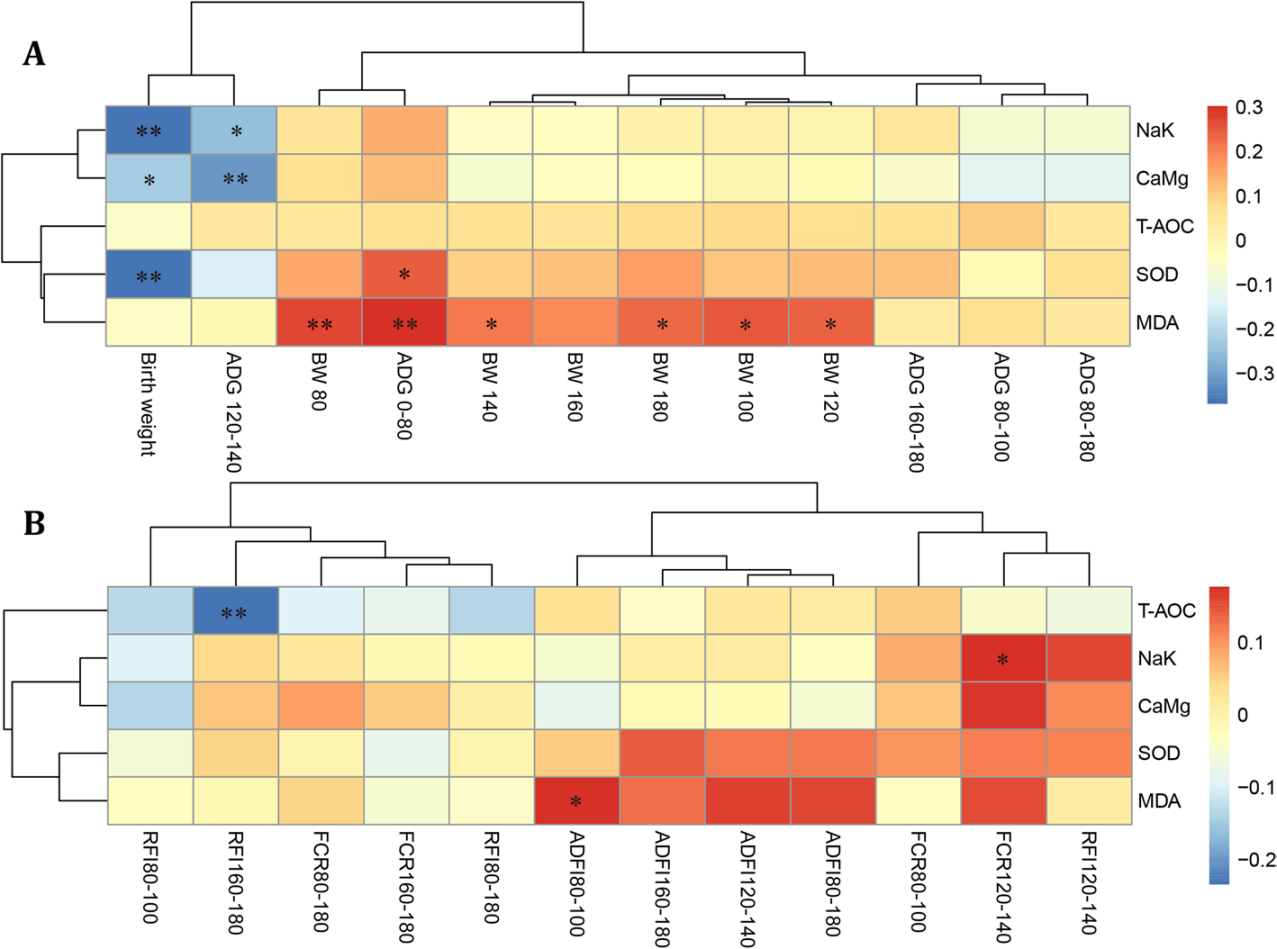
Correlation analysis of intestinal ATPase activity and antioxidant indexes with growth traits and feed efficiency of Hu sheep. A, Correlation analysis of intestinal ATPase activity and antioxidant indexes with growth traits of Hu sheep; B, Correlation analysis of intestinal ATPase activity and antioxidant indexes with feed efficiency of Hu sheep. The red and blue gradients indicate positive or negative correlations, respectively. ** indicates a very significant difference (*P* < 0.01), * indicates a significant difference (*P* < 0.05). T-AOC = total antioxidant capacity; SOD = superoxide dismutase; MDA = malondialdehyde; ADG = average daily gain; BW = body weight; RFI = residual feed intake; FCR = feed conversion ratio; ADFI = average daily feed intake

The correlations between growth traits, feed intake, and feed efficiency in Hu sheep with the ATPase activity and antioxidant indicators in the jejunal tissue are presented in Fig 1A. The activities of Na^+^ K^+^-ATPase, Ca^2+^ Mg^2+^-ATPase, and SOD showed significant negative correlations with birth weight (*P* < 0.05). Na^+^ K^+^-ATPase and Ca^2+^ Mg^2+^-ATPase activities correlated significantly and negatively with the daily weight gain of Hu sheep at 120–140 days old (*P* < 0.05). SOD activity exhibited a significant positive correlation with the daily weight gain at 0–80 days old (*P* < 0.05). The MDA content demonstrated an extremely significant positive correlation with the daily weight gain of Hu sheep at 0–80 days old. Furthermore, from 80 days old until the end of the 180-day trial, the MDA content correlated significantly and positively with the body weights at various stages (*P* < 0.05). In terms of feed efficiency (Fig 1B), the jejunal tissue total antioxidant capacity (T-AOC) showed a significant negative correlation with the residual feed intake at 160–180 days old (*P* < 0.05). The Na^+^ K^+^-ATPase activity exhibited a significant positive correlation with feed conversion efficiency at 120–140 days old (*P* < 0.05). The MDA content showed a significant positive correlation with the average daily feed intake at 80–100 days old (*P* < 0.05).

### Relationship between intestinal ATPase activity and MDA content on growth traits and feed conversion efficiency in Hu sheep

Based on the results of the correlation analysis, the relationship between the MDA content and antioxidant indicators and the growth and feeding traits of Hu sheep was found to be significant. Na^+^ K^+^-ATPase is considered the most crucial ATPase on the cell membrane. Therefore, in this experiment, the top 10 individuals with the highest and lowest ATPase activity and MDA content were selected, forming the H-ATPase and L-ATPase groups, H-MDA and L-MDA groups. The differences in growth traits and feed conversion efficiencies between these extreme groups were compared (Table 1). The results showed that the birth weight of Hu sheep in the L-ATP group was significantly higher than that in the H-ATP group (*P* < 0.05). At 80 d, 100 d, and 120 d, the body weight of the H-MDA group was significantly higher than that of the L-MDA group (*P* < 0.05). From 0–80 d, the ADG of the H-MDA group was significantly higher than that of the L-MDA group (*P* < 0.05). At 120–140 d, the FCR of the H-MDA group was significantly higher than that of the L-MDA group (*P* < 0.05).

**Table 1:** Effects of high and low ATPase activity and MDA content on growth performance and feed efficiency of Hu sheep.

Items	Groups		SEM	*P*-value	Groups		SEM	*P*-value
	H-ATP	L-ATP			H-MDA	L-MDA		
BW, Kg								
Birth	3.59	4.17	0.22	0.017	4.30	4.00	0.28	0.313
80 d	17.99	17.80	2.07	0.926	20.88	16.84	1.62	0.023
100 d	22.76	23.10	2.21	0.881	26.24	22.29	1.83	0.044
120 d	28.58	29.32	2.44	0.766	32.04	27.63	1.92	0.033
140 d	33.99	35.32	2.53	0.607	37.36	33.36	2.13	0.077
160 d	40.03	41.12	2.52	0.669	43.37	39.38	2.19	0.086
180 d	45.83	46.42	2.52	0.817	48.55	44.67	2.43	0.128
ADG, Kg/d								
0–80 d	0.18	0.17	0.03	0.721	0.21	0.16	0.02	0.022
80–100 d	0.24	0.27	0.03	0.321	0.27	0.27	0.03	0.880
120–140 d	0.27	0.30	0.02	0.148	0.27	0.29	0.03	0.446
160–180 d	0.29	0.27	0.03	0.419	0.26	0.27	0.03	0.900
80–180 d	0.28	0.29	0.02	0.594	0.28	0.28	0.01	0.945
ADFI, Kg/d								
80–100 d	1.01	1.09	0.12	0.486	1.17	1.06	0.10	0.284
120–140 d	1.72	1.68	0.13	0.789	1.84	1.69	0.13	0.242
160–180 d	2.02	1.92	0.13	0.446	2.04	2.00	0.13	0.752
80–180 d	1.60	1.63	0.10	0.765	1.70	1.60	0.10	0.366
FCR								
80–100 d	4.21	4.24	0.44	0.957	4.41	3.94	0.37	0.213
120–140 d	6.46	5.68	0.55	0.171	7.40	5.91	0.68	0.042
160–180 d	7.10	7.73	0.85	0.468	8.03	8.08	0.83	0.954
80–180 d	5.73	5.73	0.33	0.995	6.17	5.75	0.28	0.150
RFI, Kg/d								
80–100 d	−0.04	0.01	0.05	0.379	−0.06	0.00	0.05	0.236
120–140 d	0.04	−0.08	0.06	0.061	0.02	0.02	0.06	0.935
160–180 d	0.01	−0.10	0.12	0.366	−0.04	0.05	0.07	0.219
80–180 d	−0.02	−0.02	0.05	1.000	−0.02	0.02	0.04	0.315

*Abbreviations*: *BW* body weight, *ADG* average daily gain, *ADFI* average daily feed intake, *FCR* feed conversion ratio, *RFI* residual feed intake, *H-ATP* high ATP level group, *L-ATP* low ATP level group, *H-MDA* high MDA level group, *L- MDA* low MDA level group, *SEM* standard error of mean

### Correlation analysis of ATPase activity and antioxidant indexes in intestinal tissues with the development of internal organs of Hu sheep

**Fig. 2:**
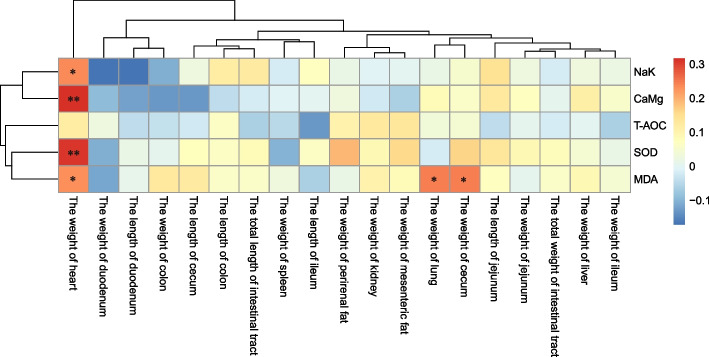
Correlation analysis of intestinal ATPase and antioxidant indexes with the internal organ development of Hu sheep. The red and blue gradients indicate positive or negative correlations, respectively. ** indicates a very significant difference (*P* < 0.01), * indicates a significant difference (*P* < 0.05). T-AOC = total antioxidant capacity; SOD = superoxide dismutase; MDA = malondialdehyde

Correlation analysis was conducted between the ATPase activity and antioxidant indicators in intestinal tissues and the visceral organ weights of Hu sheep (Fig. 2). The results indicated a significant positive correlation between the Na^+^ K^+^-ATPase, Ca^2+^ Mg^2+^-ATPase, and SOD activities in intestinal tissues and the weight of the Hu sheep heart (*P* < 0.05). Additionally, there was a significant positive correlation between MDA contents and the Hu sheep heart weight, lung weight, and cecum weight (*P* < 0.05).

### Relationship between intestinal ATPase activity and MDA content on the intestinal tract development in Hu sheep

We conducted an analysis to investigate the impact of intestinal ATPase activity and MDA content on the development of the intestinal tract in Hu sheep (Table 2). The results indicate that in the H-ATP group, the length of the ileum in Hu sheep is significantly higher than that in the L-ATP group (*P* < 0.05), with no significant impact observed on other indicators due to ATP activity (*P* > 0.05). However, in the H-MDA group, the relative duodenum weight (%body), jejunum weight, relative jejunum weight (%body), relative jejunum weight (%intestinal tract), ileum weight, and relative colon weight (%intestinal tract) are all significantly higher compared to the L-MDA group (*P* < 0.05).

**Table 2:** Effects of high and low ATPase activity and MDA content on development of intestinal tract of Hu sheep

Traits	Groups		SEM	*P*-value	Groups		SEM	*P*-value
	H-ATP	L-ATP			H-MDA	L-MDA		
Duodenum								
Weight (g)	35.35	36.20	1.779	0.739	40.22	36.95	2.598	0.386
Length (cm)	65.20	64.50	3.136	0.876	64.05	64.80	2.458	0.832
Relative weight (%body)	0.08	0.07	0.000	0.895	0.09	0.07	0.000	0.035
Relative length (%IT)	1.66	1.79	0.001	0.273	1.78	1.79	0.001	0.911
Jejunum								
Weight (g)	899.80	877.10	30.360	0.603	931.36	853.85	21.743	0.021
Length (m)	29.36	27.94	0.779	0.213	29.51	28.03	0.780	0.195
Relative weight (%body)	1.86	1.79	0.001	0.538	1.89	1.67	0.001	0.050
Relative length (%IT)	76.25	77.49	0.006	0.192	78.13	76.73	0.007	0.174
Ileum								
weight (g)	29.80	24.70	2.187	0.116	28.75	23.20	1.664	0.030
length (cm)	51.70	40.60	3.684	0.047	47.40	44.20	4.630	0.631
Relative weight (%body)	0.06	0.05	0.000	0.220	0.05	0.05	0.000	0.281
Relative weight (%IT)	2.02	1.77	0.001	0.162	2.03	1.72	0.001	0.053
Colon								
weight (g)	385.05	409.35	13.136	0.207	383.80	360.95	19.666	0.422
length (cm)	742.90	670.10	33.237	0.139	691.30	679.00	39.965	0.830
Relative weight (%body)	0.80	0.84	0.000	0.432	0.74	0.78	0.000	0.545
Relative length (%IT)	20.52	18.60	0.007	0.081	19.66	18.00	0.008	0.144
Cecum								
weight (g)	61.05	56.85	3.146	0.358	61.70	54.10	4.549	0.253
length (cm)	39.90	35.40	2.389	0.199	37.70	34.50	2.852	0.438
Relative weight (%body)	0.13	0.12	0.000	0.305	0.12	0.12	0.000	0.735
Relative length (%IT)	1.04	0.98	0.001	0.506	1.06	0.91	0.001	0.115
The total weight of IT (kg)	1.46	1.40	0.045	0.406	1.42	1.31	0.038	0.052
The total length of IT (m)	38.76	36.05	0.735	0.017	37.16	36.00	1.079	0.457

*Abbreviations*: *IT* intestinal tract, *H-ATP* high ATP level group, *L-ATP* low ATP level group, *H-MDA* high MDA level group, *L- MDA* low MDA level group, *SEM* = standard error of mean

### Relationship between ATPase activity and MDA content on the jejunum morphology in Hu sheep

We conducted a further analysis of the differences in intestinal tissue morphological indicators between the H-ATP group and L-ATP group, as well as between the H-MDA group and L-MDA group (Table 3). The results indicated that the crypt depth in the H-ATP group was significantly higher than that in the L-ATP group (*P* < 0.05), and the circular muscle thickness was significantly higher than that in the L-ATP group (*P* < 0.05). Additionally, in the H-MDA group, the villus height, crypt depth, and longitudinal muscle thickness were significantly higher than those in the L-MDA group. There were no significant differences observed for the other indicators.

**Table 3:** Effects of high and low ATP and MDA on jejunum tissue morphology of Hu sheep

Items	Groups		SEM	*P*-value	Groups		SEM	*P*-value
	H-ATP	L-ATP			H-MDA	L-MDA		
VH, µm	813.58	807.23	27.46	0.818	910.78	733.52	36.09	0.000
VW, µm	288.01	260.45	18.85	0.149	272.42	269.42	22.71	0.896
CD, µm	577.13	530.66	18.01	0.012	589.31	472.99	18.80	0.000
AMT, µm	285.57	223.00	13.23	0.000	276.64	267.10	20.09	0.636
LMT, µm	86.08	76.91	5.51	0.101	102.00	86.46	4.89	0.002
VH:CD, %	142.94	153.33	5.70	0.073	155.67	158.22	7.44	0.732
VW:CD, %	50.20	50.02	3.65	0.961	47.30	58.37	5.10	0.034

*Abbreviations*: *VH* Villus height, *VW* Villus width, *CD* Crypt depth, *AMT* Annular muscle thickness, *LMT* Longitudinal muscle thickness, *VH:CD* Villus height: Crypt depth, *VW:CD* Villus width: Crypt depth, *H-ATP* high ATP level group, *L-ATP* low ATP level group, *SEM* standard error of mean, *H-MDA* high MDA level group, *L- MDA* low MDA level group

### Relationship between ATPase activity and MDA content on jejunal microbial diversity in Hu sheep

**Fig. 3:**
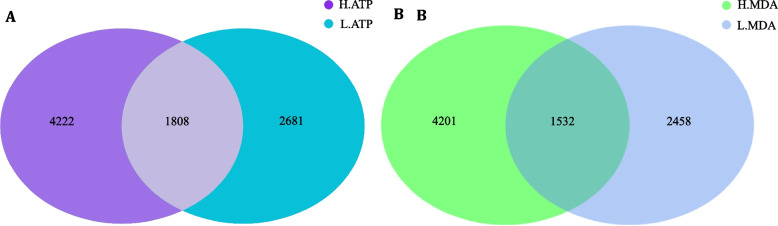
Relationship between ATPase activity and MDA content on jejunal microbial diversity in Hu sheep. A, amplicon sequence variants (ASVs) of H-ATP (high ATP) and L-ATP (low ATP) group of Hu sheep; B, ASVs of H-MDA (high malondialdehyde) and L-MDA (low malondialdehyde) groups of Hu sheep

To investigate the impact of jejunum tissue ATPase activity and antioxidant function on the diversity of the microbial community in Hu sheep, this experiment employed 16s rRNA amplicon sequencing technology to compare the differences in the gut microbiota between the high H-ATP and low L-ATP groups, as well as between the high H-MDA and low L-MDA groups. After filtering and quality control of the raw data, 74,568 to 141,026 effective sequences were obtained, with Q20 and Q30 high quality data exceeding 97.41% and 91.75%, respectively. The statistical results from the data processing are presented in Supplementary Tables S2 and S3. Dilution curves indicated ample sequencing depth, meeting the requirements for subsequent data analysis (Supplementary Fig. S1). As shown in Fig. 3A, 8711 ASVs were detected collectively in the H-ATP and L-ATP groups, with only 1808 ASVs shared between the two groups, and the unique ASVs in each group were 2681 and 4222, respectively. Similarly, Fig. 3B shows that 8191 ASVs were detected collectively in the H-MDA and L-MDA groups, with only 1532 ASVs shared between the two groups. The H-MDA group had 2458 unique ASVs, while the L-MDA group had 4201 unique ASVs.

**Fig. 4:**
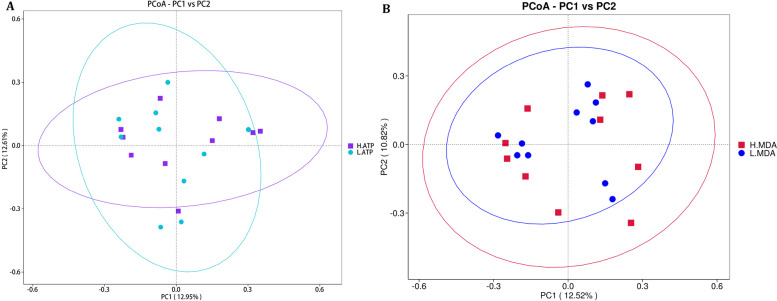
Relationship between ATPase activity and MDA content on jejunal microbial Beta diversity in Hu sheep. A, Analysis of Beta diversity of jejunum microorganisms in Hu sheep by H-ATP (high ATP) group and L-ATP (low ATP) group; B, Analysis of Beta diversity of jejunum microorganisms in Hu sheep by H-MDA (high malondialdehyde) group and L-MDA (low malondialdehyde) group. PCoA = principal coordinate analysis

The results of alpha diversity analysis (Table 4) indicated a significant increase in both observed features and Chao1 index in the L-ATP group compared with those in the H-ATP group (*P* < 0.05). Furthermore, a noticeable upward trend was observed in the L-MDA group concerning observed features and Chao1 index compared with that in the H-MDA group, although this difference fell just outside the conventional significance threshold (0.05 < *P* < 0.1). However, PCoA analysis revealed no distinct clustering based on weighted unifrac measurements (Fig. 4A-B). Additionally, ANOSIM analysis using the Bray–Curtis metric demonstrate an non-significant difference between the groups (*P* > 0.05).

**Table 4:** Abundance and diversity index of jejunal microbiota of Hu sheep

Diversity indices	Groups		SEM	*P*-Value	Groups		SEM	*P*-Value
	H-ATP	L-ATP			H-MDA	L-MDA		
Chao1 index	680.06	928.76	113.36	0.042	650.85	902.65	128.86	0.066
Observed index	657.50	898.20	112.34	0.046	621.70	863.00	124.71	0.069
Shannon index	5.05	5.65	0.77	0.442	4.83	5.70	0.65	0.193
Simpson index	0.82	0.87	0.08	0.572	0.81	0.90	0.07	0.254

*Abbreviations*: *H-ATP* high ATP level group, *L-ATP* low ATP level group, *SEM* standard error of mean, *H-MDA* high MDA level group, *L- MDA* low MDA level group

### Relationship between ATPase activity and MDA content on the jejunal microbial composition in Hu sheep

**Fig. 5:**
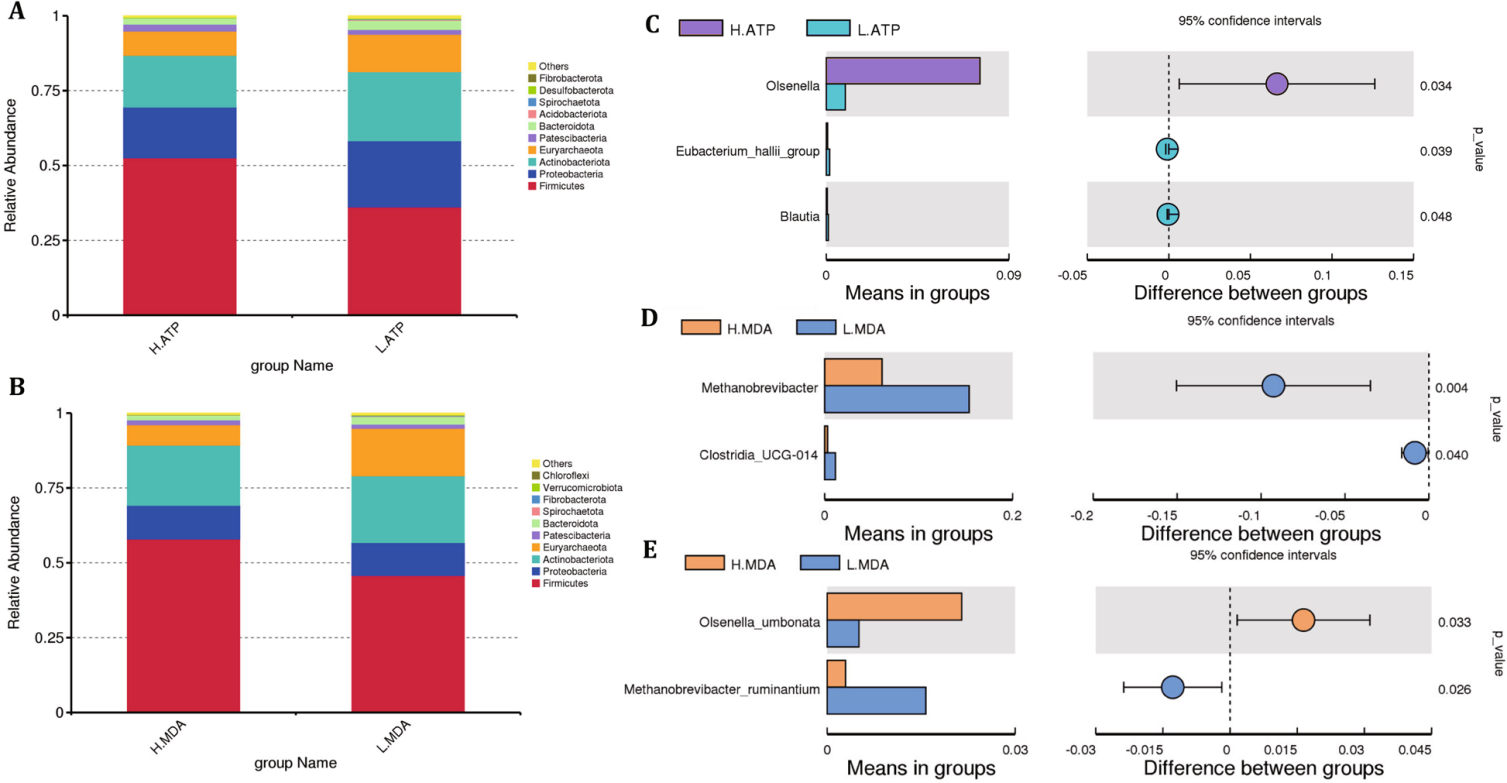
Relationship between ATPase activity and MDA content on jejunal microbial composition in Hu sheep. A-B, relative abundances of high and low ATPase and MDA content at the phylum level. C, Analysis of species differences between T-test groups with high and low ATPase at genus level. D-E, Analysis of species differences between T-test groups of high and low MDA at genus and species levels. H-ATP = high ATP; L-ATP = low ATP; H-MDA = high malondialdehyde; L-MDA = low malondialdehyde

At the phylum level, the predominant microbial taxa in the jejunum (relative abundance > 5%) were Firmicutes, Proteobacteria, Actinobacteriota, and Euryarchaeota in each group (Fig. 5A-B) Among the top 10 most abundant phyla, the relative abundance of Euryarchaeota in the H-MDA group was significantly lower than that in the L-MDA group (*P* < 0.05), while there were no significant differences in the relative abundance of the other phyla (*P* > 0.05). At the genus level (Fig. 5C-D), the relative abundance of *Olsenella* in the H-ATP group was significantly higher than that in the L-ATP group (*P* < 0.05), while *Eubacterium hallii group* and *Blautia* showed significantly lower relative abundance in the H-ATP group (*P* < 0.05). In the H-MDA group, the relative abundances of *Methanobrevibacter* and *Clostridia UCG-014* were significantly lower than those in the L-MDA group (*P* < 0.05). At the species level (Fig. 5E), the relative abundance of *Olsenella umbonata* in the H-MDA group was significantly higher than that in the L-MDA group (*P* < 0.05), while the abundance of *Methanobrevibacter ruminantium* was significantly lower than that in the L-MDA group (*P* < 0.05).

### In vitro validation of the interaction between Olsenella umbonata and MDA

**Fig. 6:**
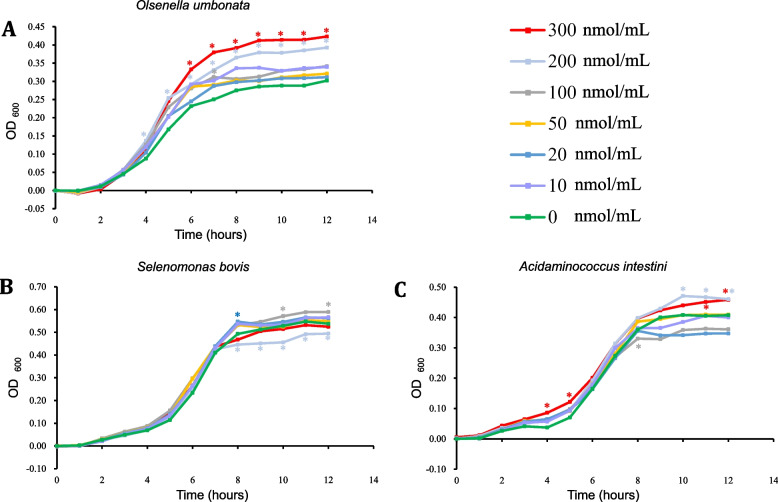
Effects of different malondialdehyde (MDA) concentrations on the anaerobic growth of *Olsenella umbonata, Selenomonas bovis* and *Acidaminococcus intestini* in vitro

To directly validate the interactions between *Olsenella umbonata* and MDA, the anaerobic growth of *Olsenella umbonata* and two other common intestinal bacteria was characterized at different concentrations of MDA. The results indicated that with increasing MDA concentrations, the OD_600_ of *Olsenella umbonata* was higher after 4 hours of cultivation, demonstrating that MDA indeed promoted the proliferation of *Olsenella umbonata* ((Fig. 6A). By contrast, the anaerobic cultivation results for *Selenomonas bovis* and *Acidaminococcus intestini* (Fig. 6B-C) showed inconsistent effects of different MDA concentrations on the growth curves of these two species, with specific MDA concentrations inhibiting their proliferation (*P* < 0.05).

## Discussion

The small intestine serves as the primary organ for nutrient digestion and absorption and is in direct contact with toxins and metabolites produced by intestinal bacteria. Therefore, the implications of changes to intestinal integrity and function on overall health should not be underestimated [34]. ATPase and antioxidant function in intestinal tissue are important indicators of intestinal metabolic strength [21, 30]. Under normal physiological conditions, intestinal epithelial cells exhibit exceptionally high metabolic activity, which is essential for the function and development of intestinal tissue. However, this metabolic process also generates a significant amount of oxygen free radicals [15]. Oxidative stress conditions exacerbate this process, leading to a substantial increase in the production of oxygen free radicals in the intestine. This increased production can adversely affect the growth, development, and normal metabolic functions of animals [35]. Previous analysis have shown that oxidative stress can alter serum indexes, affecting normal metabolic function and overall development in piglets [36]. Similarly, studies on calves have shown that intestinal oxidative stress resulting from early weaning not only delays rumen development, but also can lead to diarrhea and increased mortality [37]. Despite these findings, the underlying reasons for the interindividual variation in ATPase activity and the antioxidant capacity of ovine intestinal tissue, and its impact on the growth traits of sheep remain unclear.

Significantly, this study showed that the activities of Na^+^ K^+^-ATPase, Ca^2+^ Mg^2+^-ATPase, and SOD correlated negatively with the birth weight of Hu sheep, indicating that these activities are influenced by congenital factors. Lambs with higher birth weights exhibited lower activities of Na^+^ K^+^-ATPase, Ca^2+^ Mg^2+^-ATPase, and SOD. This may be linked to nutrient distribution during the embryonic period, and the resulting interindividual differences endured throughout the entirety of the experiment. During the embryonic period, the intestinal tract is inactive, leading individuals with lower intestinal metabolic activity to reduce their nutrient consumption, consequently resulting in increased body weight. In postnatal growth stages, Na^+^ K^+^-ATPase and Ca^2+^ Mg^2+^-ATPase activities were negatively correlated with ADG and positively with FCR at 120–140 days old. This may be related to the excessive consumption of absorbed nutrients by the intestinal tissues of individuals with high metabolic activity. Research indicates that approximately one-third of dietary nutrients undergo first-pass metabolism in the gut [38]. Most of the intercepted essential amino acids are utilized by intestinal tissues for catabolism through transamination and decarboxylation, producing ATP, and serving as the foundation for the synthesis of new molecules [39–41]. While individuals with high intestinal ATPase activity enjoy advantages in terms of nutrient absorption, the active metabolism leads to higher nutrient consumption. In addition, the results of this experiment indicate that individuals with higher intestinal ATPase activity and MDA content exhibit better development of the intestinal tract, with relatively greater lengths and weights. This finding further supports the perspective that individuals with higher intestinal ATPase activity intercept and consume more nutrients for intestinal tissue development. However, daily gain at 0–80 days old correlated significantly and positively with SOD activity and the MDA content. This suggested that individuals producing more oxygen-free radicals in the gut have an advantage in terms of nutrient digestion and absorption. This could be one of the reasons for the postnatal compensatory growth observed in individuals with low birth weight[42, 43].

The increased production of oxygen-free radicals in individuals with heightened metabolic activity in the gut might result in oxidative damage and the enrichment of MDA in intestinal tissue. This condition could have adverse effects on intestinal development and its barrier function. In this study, despite the accelerated growth observed in individuals with a high MDA content during 0–80 days, leading to a significantly higher body weight after 80 days, there was no significant difference in the ADG between individuals with high and low MDA levels after 80 days old. This aligns with previous findings and suggests a potential negative impact attributed to the accumulation of intestinal oxidative damage [44]. Furthermore, this study revealed a significant negative correlation between RFI and intestinal T-AOC in the late fattening period (160–180 days old). Consequently, enhancing the antioxidant capacity of intestinal tissue through nutritional interventions might foster the development of growth potential in individuals with robust intestinal tissue metabolism.

Our experiment revealed notable positive correlations between Na^+^ K^+^-ATPase, Ca^2+^ Mg^2+^- ATPase, SOD activities, and the MDA content in intestinal tissue with the heart weight. Furthermore, the MDA content correlated significantly and positively with the lung weight. These findings offer insights into the sources of the individual variations in ATPase activity and antioxidant function in sheep, highlighting the multifaceted nature of these physiological attributes. This might be partially explained by the pivotal roles played by the heart and lungs as primary oxygen-supplying organs that transport oxygen to the intestines via the circulatory system. Larger hearts and lungs lead to a more efficient oxygen supply, resulting in elevated ATPase activity. Simultaneously, increased ROS levels contribute to enhanced SOD activity and MDA contents. These outcomes also imply that intestinal metabolic activity and antioxidant function are influenced by a multitude of factors, with a particularly close connection to the circulatory system. Research has shown that the intestines are one of the most sensitive tissues and organs to ischemia-reperfusion injury [45]. Disruption of normal cellular homeostasis by ROS produced within the gastrointestinal tract might result in cardiovascular diseases [23].

The interaction between the intestinal microbiome and the host is a dynamic and complex relationship that significantly influences various aspects of animal physiology, making it a critical area of livestock research. Oxygen homeostasis has emerged as one of the mechanisms through which the host and gut microbes interact. The intestine is characterized by a distinctive oxygenation profile, with a steep gradient between the physiological hypoxic epithelial surface and the anaerobic lumen, which favors the dominance of obligate anaerobes [46]. The ATP consumed by the small intestine is primarily derived from aerobic respiration and oxidative phosphorylation. Consequently, the regulation of epithelial oxygen consumption plays a crucial role in determining the oxygen balance at the interface between the host and its environment. These intricate interplays among the microbiota, the epithelial barrier, and nutrients are also contingent upon oxygen homeostasis at the epithelial barrier. Furthermore, microbiota-derived metabolites influence oxidative phosphorylation [47], nuclear receptors [48], and other functions related to metabolism at the intestinal epithelial barrier [49]. In this study, we observed that, under the same feeding conditions, individuals with higher ATP enzyme activity and MDA content in the jejunum tissue exhibited lower intestinal microbiota diversity and richness. A study indicated that higher oxygen levels in the intestinal tract favor the proliferation of facultative anaerobes such as enterobacteria, enterococci, and streptococci, underscoring the influence of oxygen levels on the gut microbiota composition [50], Consequently, our findings suggested that individuals with high ATPase activity and MDA content might have disrupted intestinal oxygen homeostasis through intense aerobic respiration, thus inhibiting certain microbial species and reducing intestinal microbial diversity. Previous research has generally demonstrated that a higher diversity of the gastrointestinal microbiota correlates with increased resilience, resistance, and stability of the microbial ecosystem in the face of environmental changes [51, 52]. Nonetheless, other studies have indicated that the premature development and diversification of the microbiota might be detrimental to immune function [53, 54]. The mechanism by which high ATPase activity and a reduced MDA content in intestinal tissue decrease microbial diversity warrants further investigation.

While this study revealed significant influences of ATPase activity and the MDA content on the Chao1 index and observed species, their effect on Beta diversity was not pronounced, primarily because of their relatively minor impact on the dominant bacterial taxa. Nonetheless, among the taxa with higher relative abundances, we observed changes in the abundance of certain specific taxa. The relative abundance of *Euryarchaeota* was lower in individuals with high ATP enzyme activity and MDA contents, and the relative abundance of *Methanobrevibacter* was lower in individuals with a high MDA content. *Euryarchaeota* represents a major branch of methane-producing archaea capable of converting acetates, methanol, and methylamines within the intestinal tract into methane [55, 56], simultaneously generating ATP [57]. *Methanobrevibacter* is an important methane-producing archaeal genus exhibiting extreme anaerobic characteristics [58]. The significant fluctuations in its abundance are likely attributable to the aforementioned differences in intestinal oxygen homeostasis among individuals with varying ATP enzyme activity and MDA contents. Although studies have indicated that the intestinal oxygenation profile can influence the composition of the gut microbiota, the impact of intestinal oxygen homeostasis on archaea has not been comprehensively explored. However, it is inferred that enhancing intestinal ATP enzyme activity and antioxidant function might reduce methane emissions and alleviate environmental pressures by decreasing the abundance of methane-producing archaea in the gut.

Furthermore, although the results of this experiment indicated that high ATP enzyme activity and MDA content decreased intestinal microbiota diversity and reduced the abundance of specific taxa, we observed a substantial increase in the relative abundance of *Olsenella umbonata* in individuals with high MDA levels compared to those with low MDA levels. *Olsenella* is a dominant genus in the jejunum chyme [59] and plays a crucial role in host nutritional metabolism and maintaining intestinal balance [35]. Our results suggested that *Olsenella umbonata* might possess specific adaptive mechanisms related to intestinal oxygen homeostasis. Research has shown that aldehydes exhibit antimicrobial properties against various microorganisms [60]. However, certain bacteria were observed to exhibit aldehyde resistance [61]. The differential adaptability of various bacteria to MDA, an aldehyde compound, might be a factor through which the host’s intestinal cell aerobic respiration intensity and antioxidant capacity interact with and affect the microbial community structure. To validate this hypothesis, we conducted *in vitro* anaerobic culture experiments to investigate the impact of the MDA concentration on the growth curves of *Olsenella umbonata* and two other common intestinal bacterial species. The results confirmed that MDA promotes the proliferation of *Olsenella umbonata*, with a more pronounced effect at higher MDA concentrations. Additionally, a certain concentration of MDA inhibited the proliferation of the other two common bacterial species, highlighting the unique adaptive mechanism of *Olsenella umbonata* to MDA. *Olsenella umbonata* might reduce the MDA content in intestinal tissues through degradation, thereby mitigating the adverse effects of MDA accumulation on intestinal cell metabolism. However, further research is required to fully understand the adaptive mechanisms of this bacterial species to MDA and its potential applications.

The findings from this study have several implications for understanding the intricate relationship between intestinal ATPase activity, antioxidant function, and various physiological aspects in Hu sheep. The strong correlation observed between jejunum ATPase and SOD activities and the initial weight of Hu sheep suggests a potential link between congenital factors and these enzymatic activities. Furthermore, the connection between ATPase activity, antioxidant performance, and growth, heart development, and intestinal morphology emphasizes the multifaceted roles of these factors in overall physiological well-being. Additionally, the impact of high ATPase activity and MDA levels on jejunum microbial diversity and specific bacterial taxa sheds light on the potential host-microbiota interaction. The confirmation of MDA’s influence on the proliferation of certain bacterial species in vitro adds depth to the understanding of these interactions. However, the study acknowledges the need for further research to elucidate individual variations in intestinal ATPase activity and antioxidant capacity. Additionally, exploration is needed into the specific mechanisms through which ATPase activity and antioxidant function influence intestinal weight and morphology, as well as the adaptive mechanisms of specific bacterial species to ATPase and MDA. These research pursuits will collectively contribute to a more comprehensive understanding of these complex relationships.

## Conclusion

This study revealed that lambs with higher birth weights exhibit lower Na^+^ K^+^-ATPase, Ca^2+^ Mg^2+^-ATPase, and SOD activities. The ATPase activity and antioxidant capacity in intestinal tissue are closely associated with growth performance at specific ages, as well as heart development and intestinal morphology. High ATPase activity and MDA levels decreased jejunum microbial diversity and affected the abundance of specific bacteria. *In vitro* experiments confirmed the influence of the MDA content on the proliferation of certain species, indicating a potential interaction between the host and its intestinal microbiota. However, further research is needed to fully explain the individual variations in intestinal ATPase activity and antioxidant capacity, as well as the adaptive mechanisms of specific bacterial species to ATPase and MDA.

## Methods

### Experimental design

**Fig. 7:**
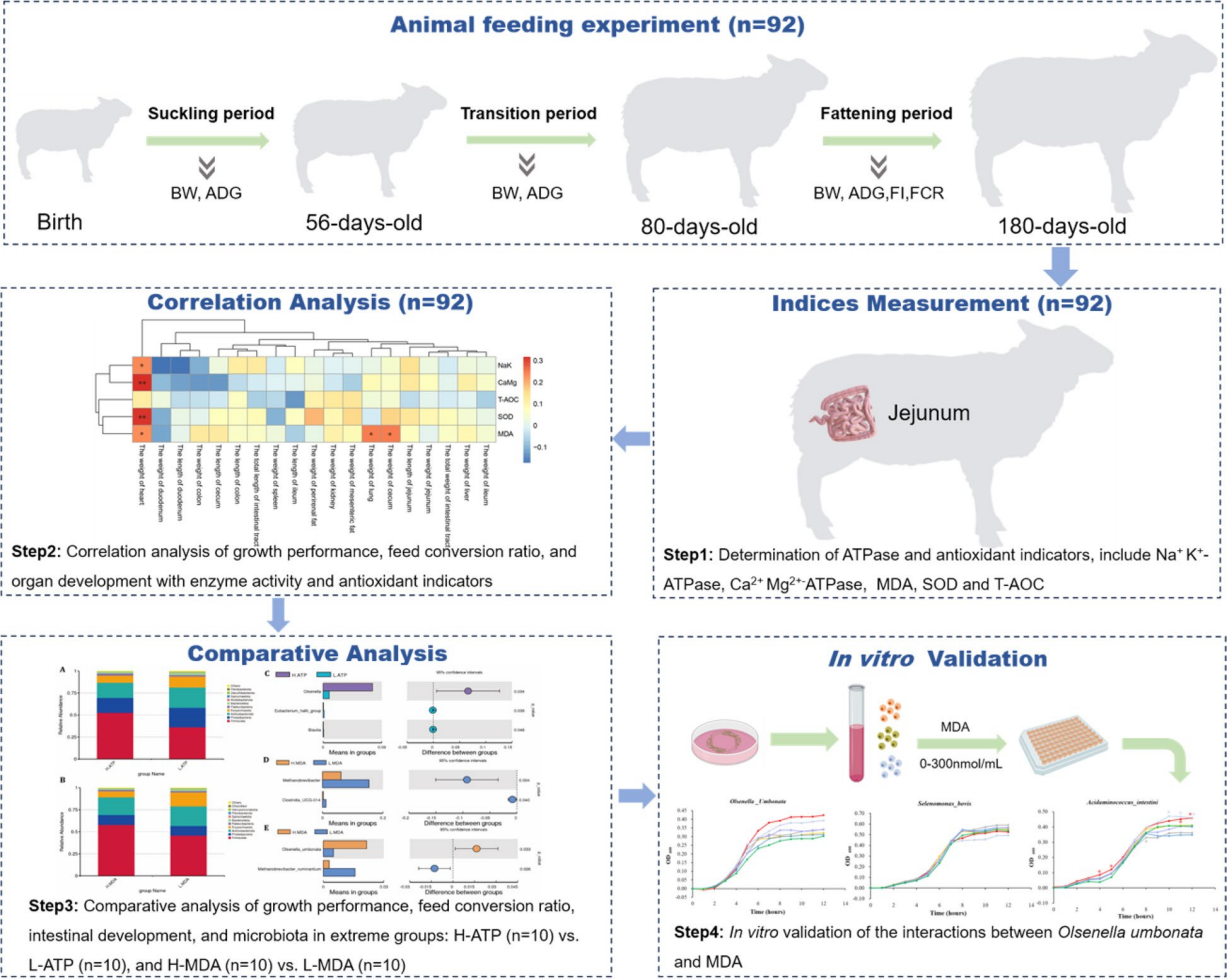
The schematic diagram of study design and workflow

A total of 92 healthy male Hu sheep lambs with similar birthdates (Birth weight: 4.02 ± 0.10 kg) were randomly selected for the experiment. The sheep used in this study were provided by Defu Agriculture Co. Ltd. (Minqin, China). The feeding trial spanned from birth to 180 days of age, after which all lambs were slaughtered. Measurements were taken for the activities of Na^+^ K^+^-ATPase, Ca^2+^ Mg^2+^-ATPase, SOD, T-AOC, and MDA content in the jejunum tissue. The study analyzed the correlation between these enzyme activities and antioxidant indicators with various parameters including growth performance, development of visceral organs, intestinal tissue, and the jejunal microbiota. Based on the analysis of ATPase and MDA values, the top 10 individuals with the highest and lowest Na^+^ K^+^-ATPase activity, as well as the top 10 individuals with the highest and lowest MDA content, were selected. This selection formed the High ATPase group (H-ATP, *n* = 10) and Low ATPase group (L-ATP, *n* = 10), High MDA group (H-MDA, *n* = 10), and Low MDA group (L-MDA, *n* = 10). Among these, four sheep exhibited both the highest ATPase activity and MDA content, while three sheep exhibited both the lowest ATPase activity and MDA content. Subsequent comparisons were made between these extreme groups to evaluate differences in growth traits, feed conversion efficiencies, jejunum morphology indices, and jejunal microbiota. The experimental design is shown in Fig. 7.

### Animal husbandry and sample collection

All lambs were kept with their ewes before weaning, supplemented with starter feed from the age of 7 days, and subjected to a standardized immunization protocol. Lambs had *ad libitum* access to starter feed and water. Weaning occurred at 56 days old, and after being weaned, all lambs were housed individually in a 0.8-m^2^ pen so that all measurements could be performed individually. All the lambs were housed under the same management conditions. A 14-day transition period followed weaning, during which the diet transitioned from starter feed to a total mixed pellet fattening feed. The starter feed and total mixed pellet fattening feed were produced by Gansu Runmu Biological Engineering Co., Ltd. (Jinchang, Gansu, China), and the formulation and nutritional composition are detailed in Supplementary Material Table S1. Subsequently, after a 10-day preliminary trial period, lamb body weight (BW) and feed intake (FI) were measured every 20 days until the end of the trial (180 days old). During the experimental, the coefficient of the linear regression of BW was used to calculate the average daily gain (ADG); The metabolic body weight (MBW) reference the method of Basarab [62].The feed conversion ratio (FCR) according to the following equation: FCR = FI / (BW_180_-BW_80_). Additionally, a linear regression model was used to calculate the residual feed intake (RFI), incorporating the dry matter intake (DMI), ADG, and MBW data for all sheep [63, 64]. The linear regression model can be expressed as: Y_*j*_ = β_0_ + β_1_ (MBW_*j*_) + β_2_ (ADG_*j*_) +e_*j*_. In this formula: Y_*j*_ represents the actual average DMI of the *j* animal, β_0_ represents the partial regression intercept, β_1_ represents the partial regression coefficient on MBW, β_2_ represents the partial regression coefficient on ADG, and e_*j*_ represents a vector of random residuals.

At 180 days old, all the experimental lambs were slaughtered within three days according to the standard protocols. Following a 12-hour fasting period from both feed and water, the lambs were weighed and then transported to the experimental abattoir, where they were slaughtered by severing their jugular veins and carotid arteries. Immediately after slaughter, the duodenum, jejunum, ileum, colon, and cecum were carefully separated, emptied of their contents, and rinsed with physiological saline. The weights and lengths of each section of the intestinal tract were then measured. The percentage representation of each specific intestinal segment relative to body weight and total intestinal length was calculated from these measurements. The computational formula is articulated as follows: relative weight (%body) = intestinal tract weight / premortem body weight; relative length (%intestinal tract) = intestinal tract length / total intestinal tract length.

The contents of the proximal jejunum were homogenized, and 5 mL of the homogenized jejunal contents were collected aseptically into sterile cryovials, and stored at -20 °C for subsequent 16s rRNA sequencing of the jejunal microbiota. Tissue samples were obtained from the proximal jejunum, specifically from a segment located 0.5 to 1.0 m posterior to the end of the duodenum. Each 0.5 m segment was cut into two equidistant subsamples and rinsed in ice-cold saline. The first subsample was collected into sterile cryovials, immediately immersed in liquid nitrogen, transported to the laboratory, and stored at -80 °C for the subsequent determination of ATPase activity and antioxidant indices. The second subsample was collected and preserved in 4% paraformaldehyde for histological analysis after fixation.

### Determination of ATPase activity and the antioxidant index

The collected jejunum tissue samples were ground in liquid nitrogen, the tissue weight was determined accurately, and 9 x the volume of normal saline was added according to a ratio of weight (g) to volume (mL) = 1:9. The supernatant was obtained by centrifugation at 3000rpm/min for 10min and a 10% tissue homogenate was prepared for measurement. ATPase activity, TP (Total Protein) concentration, and antioxidant index detection kits were procured from the Institute of Biotechnology, Jiangsu Jiancheng Bioengineering Research Institute (Nanjing, Jiangsu, China). Each parameter was rigorously measured following the methods outlined in the respective kit instructions, utilizing a Thermo Scientific™ Varioskan™ LUX multimode microplate reader (Thermo Fisher Scientific, Vantaa, Finland) to measurement the absorbance.

### Intestinal tissue morphometry

For morphological observation, jejunum tissue samples were embedded in paraffin and cut into 5 µm-thick sections, stained with hematoxylin-eosin (HE), and observed under an optical microscope (BA210 Digital, Motic, Hong Kong, China). Five well-developed and well-oriented villi were selected from each jejunal tissue section for observation, and the villus height, villus width, crypt depth, annular muscle thickness, and longitudinal muscle thickness were determined using an image analysis system (Motic Image Plus 2.0, Motic China Group Co. Ltd., Xiamen, China) for intestinal morphology analysis.

### 16s rRNA gene sequencing

DNA was extracted from the intestinal content samples using a Magnetic Stool DNA Kit (TianGen, Beijing, China, Catalog #: DP712). The DNA concentration and purity were monitored using 1% agarose gels. According to the concentration, DNA was diluted to 1ng/µL using sterile water. PCR amplification was performed using primers in the target region 16SV3-V4 (515-F: CCTAYGGGRBGCASCAG and 806-R: GGACTACNNGGGTATCTAAT). All PCR reactions were carried out with 15 µL of Phusion® High -Fidelity PCR Master Mix (New England Biolabs, Ipswich, MA, USA); 2 µM of forward and reverse primers; and about 10 ng of template DNA. Thermal cycling consisted of initial denaturation at 98 °C for 1 min, followed by 30 cycles of denaturation at 98 °C for 10 s, annealing at 50 °C for 30 s, and elongation at 72 °C for 30 s, and finally 72 °C for 5 min. The same volume of 1x loading buffer (containing SYB green) was mixed with the PCR products and subjected to electrophoresis through 2% agarose gels for detection. The PCR products were mixed in equal density ratios. Then, the mixed PCR products were purified using a Qiagen Gel Extraction Kit (Qiagen, Hilden, Germany). Sequencing libraries were generated using a TruSeq® DNA PCR-Free Sample Preparation Kit (Illumina, San Diego, CA, USA) following manufacturer’s recommendations, and index codes were added. The library quality was assessed on a Qubit 2.0 Fluorometer (Thermo Scientific) and an Agilent Bioanalyzer 2100 system (Agilent, Santa Clara, CA, USA). Finally, the library was sequenced on the Illumina NovaSeq platform to generate 250 bp paired-end reads.

### Bioinformatic analysis

The paired-end reads were assigned to samples based on their unique barcodes and truncated by cutting off the barcode and primer sequences. The paired-end reads were then merged using FLASH (Version 1.2.11, http://ccb.jhu.edu/software/FLASH/) [65], which merges paired-end reads when at least some of the reads overlap the read generated from the opposite end of the same DNA fragment. The merged sequences were termed raw tags. Quality filtering on the raw tags was performed using the fastp (Version 0.23.1) software to obtain high-quality clean tags [66]. The clean tags were compared with the reference database using UCHIME Algorithm to detect chimeric sequences, which were removed [67], to leave the effective tags. The effective tags were denoised using DADA2 or the deblur module in the QIIME2 software (Version QIIME2-202006) to obtain initial amplicon sequence variants (ASVs) (default: DADA2), and then ASVs with abundances less than 5 were filtered out [68]. The absolute abundance of ASVs was normalized using a standard sequence number corresponding to the sample with the least sequences. Subsequent analysis of alpha diversity and beta diversity were all performed based on the output normalized data. The diversity, richness and uniformity of the communities in the sample were then analyzed. Alpha diversity was applied to analyze the complexity of species diversity for a sample via four indices, including Observed-species, Chao1, Shannon, and Simpson. All these indices were calculated using QIIME (Version 1.7.0) and displayed using the R software (Version 2. 15.3). Beta diversity analysis was used to evaluate differences in species complexity between samples. Beta diversity using both a weighted and unweighted unique fraction metric (unifrac) was calculated using QIIME software (Version 1.9. 1). Cluster analysis was preceded by principal component analysis (PCA), which was applied to reduce the dimension of the original variables using the ade4 package and ggplot2 package in the R software (Version 2. 15.3). Principal Coordinate Analysis (PCoA) was performed to obtain and visualize principal coordinates from complex, multidimensional data. The prepared distance matrix of weighted or unweighted unifrac values among the samples was transformed into a new set of orthogonal axes, by which the maximum variation factor was demonstrated by the first principal coordinate, and the second maximum variation factor by the second principal coordinate, and so on. PCoA analysis was displayed by the ade4 package and ggplot2 package in the R software. Unweighted Pair-group Method with Arithmetic Means (UPGMA) Clustering was used as a hierarchical clustering method to interpret the distance matrix using average linkage, which was conducted using the QIIME software.

### In vitro growth experiments of Olsenella umbonata

The 16s rRNA sequencing results indicated a close correlation between the MDA content in intestinal tissue and the abundance of *Olsenella umbonata*. To directly validate the interactions between *Olsenella umbonata* and MDA, the anaerobic growth of *Olsenella umbonata* was characterized at different concentrations of MDA. This characterization was then compared with the anaerobic growth characteristics of two other common intestinal bacteria, *Selenomonas bovis* and *Acidaminococcus intestini,* at different MDA concentrations. These strains were previously isolated from rumen contents and feces of sheep, Strain isolation involved spreading rumen contents or feces on Gifu anaerobic medium (GAM) agar plates, followed by incubation in an anaerobic workstation (Maworde-biotech, Beijing, China). Colonies were then selected for further isolation and culture, and 16S rDNA sequencing was used for identification. For cultivation and growth curve determination, *Olsenella umbonata*, *Selenomonas bovis*, and *Acidaminococcus intestini* strains were cultured in GAM supplemented with MDA at 0, 10, 20, 50, 100, 200, and 300 nmol/mL, respectively, with four replicates for each concentration. Cultivation and growth curve determination were performed in an anaerobic workstation (Maworde-biotech, Beijing, China), and optical density at 600 nm was measured at intervals of 1 h using the Stratus microbial growth curve analyzer (Longfujia, Beijing, China).

### Statistical analysis

Spearman correlation coefficients were calculated to examine the associations between intestinal ATPase and antioxidant indexes, and growth traits and feed efficiency, as well as internal organ development. This analysis was performed using the R software (https://www.R-project.org/). A t-test was applied to assess disparities in growth traits, feed efficiency, and morphological indices of intestinal tissue between two groups, using SPSS software (Version 26.0; IBM Corp., Armonk, NY, USA). using the Bray–Curtis of ANOSIM analysis the significant between groups. To determine the significantly different species at each taxonomic level, t-test analyses were conducted using the R software (Version 4.1.1). Statistical significance was established at a threshold of *P* < 0.05.

### Supplementary Information

Supplementary Material 1: Fig. S1 16s rRNA sequencing dilution curve of ATP and MDA groups. A, 16s rRNA sequencing dilution curve of ATP groups. B, 16s rRNA sequencing dilution curve of MDA groups. ATP = adenosine triphosphate; MDA = malondialdehyde. Table S1 Dietary formulation and nutrient level (air-dry basis). Table S2 ATP group 16s rRNA sequencing data. Table S3 MDA group 16s rRNA sequencing data.

## Data Availability

Sequence files associated with each sample have been submitted to the NCBI Sequence Read Archive (SRA accession number: PRJNA1047777; Public).

## Abbreviations

ADG: Average daily gain
ADP: Adenosine diphosphate
ATP: Adenosine triphosphate
BW: Body weight
DMI: Dry matter intake
FCR: Feed conversion ratio
FI: Feed intake
MBW: Metabolic body weight
MDA: Malondialdehyde
Pi: Inorganic phosphate
RFI: Residual feed intake
ROS: Reactive oxygen species
SOD: Superoxide dismutase
T-AOC: Total antioxidant capacity
TP: Total protein
