# Supplementary material for: Relationship between jejunum ATPase activity and antioxidant function on the growth performance, feed conversion efficiency, and jejunum microbiota in Hu sheep (Ovis aries)
Source: BMC Vet Res. 2024 Jun 4;20:242. doi: 10.1186/s12917-024-04100-0 (PMC11149274; doi:10.1186/s12917-024-04100-0)
Supplement: Supplementary file 1 — Supplementary Material 1: Fig. S1 16s rRNA sequencing dilution curve of ATP and MDA groups. A, 16s rRNA sequencing dilution curve of ATP groups. B, 16s rRNA sequencing dilution curve of MDA groups. ATP = adenosine triphosphate; MDA = malondialdehyde. Table S1 Dietary formulation and nutrient level (air-dry basis). Table S2 ATP group 16s rRNA sequencing data. Table S3 MDA group 16s rRNA sequencing data. [file 12917_2024_4100_MOESM1_ESM.docx]

**Effects of jejunum ATPase activity and antioxidant function on the growth performance, feed conversion efficiency, and intestinal flora of Hu sheep (*Ovis aries*)**

Zhanyu Chen^1^, Guoxiu Wang^1^, Weimin Wang^2^, Xiaojuan Wang^1^, Yongliang Huang^1^, Jiale Jia^1^, Qihao Gao^1^, Haoyu Xu^1^, Lijuan He^1^, Yunfei Xu^1^, Zhen Liu^1^, Jinlin Sun^3^, Chong Li^1*^

**Supplementary material**


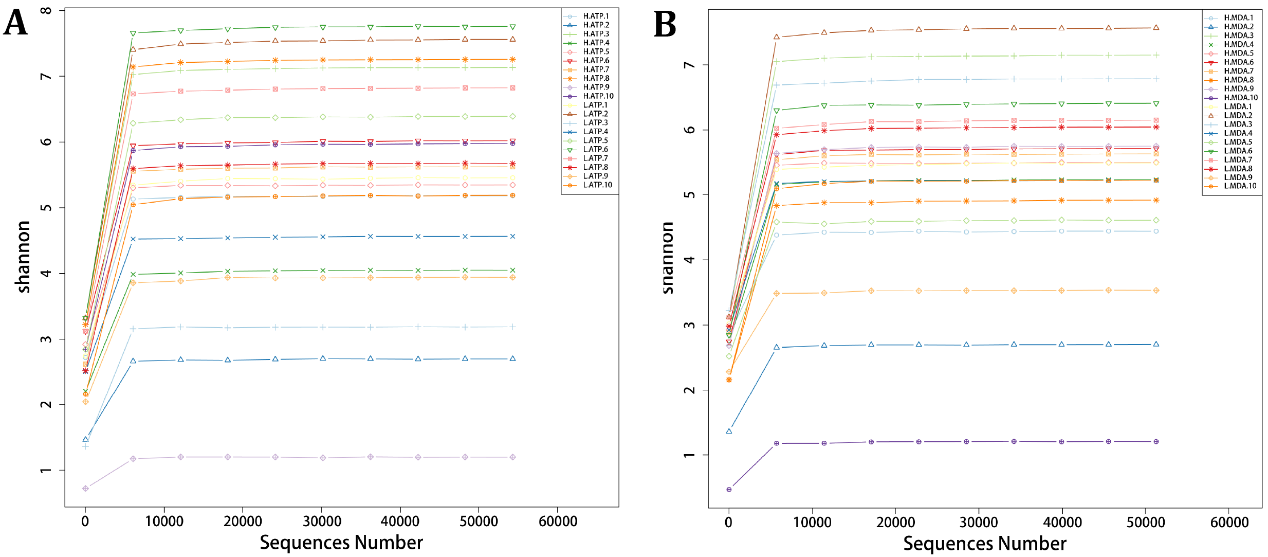


**Fig. S1** 16S rRNA sequencing dilution curve of ATP and MDA groups. A, 16S rRNA sequencing dilution curve of ATP groups. B, 16S rRNA sequencing dilution curve of MDA groups. ATP = adenosine triphosphate; MDA = malondialdehyde.

**Table S1** Dietary formulation and nutrient level (air-dry basis).

| Items | Starter | Basic diet |
| --- | --- | --- |
| **Ingredients composition (% as fed)** | | |
| Bran | 6.00 | / |
| Alfalfa meal | 18.50 | / |
| Extruded corn | 22.30 | / |
| Extruded soybean | 4.00 | / |
| Corn gluten meal | 5.00 | / |
| Limestone | 0.30 | / |
| Corn | 21.00 | 32.5 |
| Premix | 1.00 | 0.50 |
| NaCl | 0.40 | 0.70 |
| Soybean meal | 21.50 | 5.00 |
| Corn germ meal | / | 18.00 |
| Corn stalks | / | 12.00 |
| Corn hulls | / | 11.20 |
| Corn cob | / | 8.00 |
| Cotton meal | / | 5.00 |
| Molasses | / | 3.30 |
| Stone powder | / | 0.80 |
| Expanded Urea | / | 0.50 |
| Bentonite | / | 1.50 |
| Baking soda | / | 1.00 |
| Total | 100.00 | 100.00 |
| **Chemical composition (%)** | | |
| Dry matter | 90.96 | 88.78 |
| Digestible energy (MJ·kg^-1^) | 13.01 | 11.11 |
| Crude protein | 19.50 | 13.09 |
| Fat | 1.33 | 1.72 |
| Neutral detergent fiber | 18.87 | 27.08 |
| Acid detergent fiber | 8.60 | 13.09 |
| Starch | 33.10 | / |
| Crude fiber | / | 9.78 |
| Nitrogen free extract | / | 55.05 |

Notes: The premix included the following per kg of the diet: 25 mg Fe as FeSO_4_·H_2_O; 40mg Zn as ZnSO_4_·H_2_O; 8mg Cu as CuSO_4_·5H_2_O; 40mg Mn as MnSO_4_·H_2_O; 0.3 mg I as KI; 0.2 mg Se as Na_2_SeO_3_; 0.1 mg Co as CoCl_2_; 940 IU vitamin A; 111 IU vitamin D; 20 IU vitamin E, and; 0.02 mg vitamin B_12_.

**Table S2** ATP group 16S rRNA sequencing data.

| Sample | Raw reads | Clean reads | Base(nt) | GC | Q20 | Q30 |
| --- | --- | --- | --- | --- | --- | --- |
| H.ATP.1 | 116824 | 109767 | 30415953 | 55.91% | 97.75% | 92.92% |
| H.ATP.2 | 145013 | 138915 | 56073474 | 53.76% | 97.84% | 93.22% |
| H.ATP.3 | 140892 | 132306 | 38091199 | 55.92% | 97.89% | 93.34% |
| H.ATP.4 | 143458 | 134865 | 48284863 | 54.45% | 97.54% | 92.32% |
| H.ATP.5 | 131044 | 123523 | 46526909 | 56.42% | 97.83% | 93.17% |
| H.ATP.6 | 135139 | 131559 | 38854550 | 55.05% | 97.76% | 93.06% |
| H.ATP.7 | 130198 | 126610 | 42082506 | 54.80% | 97.86% | 93.22% |
| H.ATP.8 | 133241 | 129353 | 40873599 | 53.57% | 97.74% | 92.93% |
| H.ATP.9 | 133248 | 127653 | 52638480 | 45.25% | 97.41% | 91.75% |
| H.ATP.10 | 133241 | 125404 | 42902760 | 53.29% | 97.71% | 92.87% |
| L.ATP.1 | 131317 | 123466 | 43659157 | 54.49% | 97.87% | 93.25% |
| L.ATP.2 | 130363 | 122410 | 43298736 | 54.31% | 97.75% | 93.02% |
| L.ATP.3 | 128248 | 122252 | 48584840 | 53.79% | 97.78% | 93.09% |
| L.ATP.4 | 130835 | 126340 | 46451471 | 54.32% | 97.96% | 93.51% |
| L.ATP.5 | 132187 | 128712 | 43924865 | 54.80% | 97.78% | 92.99% |
| L.ATP.6 | 129610 | 125994 | 37282603 | 54.20% | 97.88% | 93.28% |
| L.ATP.7 | 143146 | 138809 | 41982711 | 54.55% | 97.78% | 93.07% |
| L.ATP.8 | 80855 | 76192 | 25115731 | 55.60% | 97.52% | 92.32% |
| L.ATP.9 | 123279 | 117538 | 45794614 | 53.19% | 97.79% | 93.08% |
| L.ATP.10 | 135027 | 127506 | 44191131 | 55.42% | 97.63% | 92.61% |

Notes: GC represent GC content; Q20 represent the proportion of bases with a Phred quality score greater than 20; Q30 represent the proportion of bases with a Phred quality score greater than 30. H-ATP = high ATP level group; L-ATP = low ATP level group.

**Table S3** MDA group 16S rRNA sequencing data.

| Sample | Raw reads | Clean reads | Base(nt) | GC | Q20 | Q30 |
| --- | --- | --- | --- | --- | --- | --- |
| H.MDA.1 | 79472 | 74568 | 23790975 | 56.15% | 97.53% | 92.40% |
| H.MDA.2 | 145013 | 138915 | 56073474 | 53.76% | 97.84% | 93.22% |
| H.MDA.3 | 140892 | 132306 | 38091199 | 55.92% | 97.89% | 93.34% |
| H.MDA.4 | 134148 | 126302 | 42301328 | 55.53% | 97.70% | 92.89% |
| H.MDA.5 | 149083 | 141026 | 47791488 | 55.27% | 97.84% | 93.21% |
| H.MDA.6 | 129400 | 125666 | 38011391 | 55.37% | 97.67% | 92.77% |
| H.MDA.7 | 130198 | 126610 | 42082506 | 54.80% | 97.86% | 93.22% |
| H.MDA.8 | 129614 | 124875 | 49241606 | 47.99% | 97.46% | 92.04% |
| H.MDA.9 | 126521 | 122824 | 36803763 | 51.95% | 97.75% | 92.87% |
| H.MDA.10 | 133248 | 127653 | 52638480 | 45.25% | 97.41% | 91.75% |
| L.MDA.1 | 131317 | 123466 | 43659157 | 54.49% | 97.87% | 93.25% |
| L.MDA.2 | 130363 | 122410 | 43298736 | 54.31% | 97.75% | 93.02% |
| L.MDA.3 | 131320 | 122897 | 38973365 | 54.20% | 97.78% | 92.97% |
| L.MDA.4 | 131918 | 123145 | 39144736 | 55.72% | 97.77% | 93.00% |
| L.MDA.5 | 130986 | 125080 | 48323823 | 53.63% | 97.77% | 93.08% |
| L.MDA.6 | 132187 | 128712 | 43924865 | 54.80% | 97.78% | 92.99% |
| L.MDA.7 | 134825 | 130780 | 40138432 | 55.43% | 97.69% | 92.90% |
| L.MDA.8 | 135139 | 131559 | 38854550 | 55.05% | 97.76% | 93.06% |
| L.MDA.9 | 131763 | 126734 | 50536439 | 47.40% | 97.56% | 92.25% |
| L.MDA.10 | 135027 | 127506 | 44191131 | 55.42% | 97.63% | 92.61% |

Notes: GC represent GC content; Q20 represent the proportion of bases with a Phred quality score greater than 20; Q30 represent the proportion of bases with a Phred quality score greater than 30. H-MDA = high MDA level group; L- MDA = low MDA level group.
